# The Role of Convalescent Plasma in COVID-19: A Conclusive Post-Pandemic Review

**DOI:** 10.3390/life13122322

**Published:** 2023-12-11

**Authors:** Massimo Franchini, Daniele Focosi

**Affiliations:** 1 Department of Transfusion Medicine and Hematology, Carlo Poma Hospital, 46100 Mantua, Italy; 2 North-Western Tuscany Blood Bank, Pisa University Hospital, 56124 Pisa, Italy; daniele.focosi@gmail.com

**Keywords:** COVID-19, SARS-CoV-2, convalescent plasma, randomized controlled trials

## Abstract

COVID-19 convalescent plasma (CCP) has represented the frontline response to the COVID-19 pandemic, largely because of encouraging historical evidences in previous pandemics, biological plausibility, and the initial unavailability of targeted antivirals. Unfortunately, investigator-initiated randomized clinical trials in 2020, launched during a stressful pandemic peak, were designed mostly at addressing the main unmet need, i.e., treating critically ill hospitalized patients who were unlikely to benefit from any antiviral therapy. The failure of most of these drugs, in combination with the lack of any sponsor, led to the false belief that convalescent plasma was useless. With the relaxing pandemic stages, evidences have instead mounted that, when administered properly (i.e., within 5 days from onset of symptoms and at high titers of neutralizing antibodies), CCP is as effective as other antivirals at preventing disease progression in outpatients, and also reduces mortality in hospitalized patients. Recently, the focus of clinical use has been on immunosuppressed patients with persistent seronegativity and infection, where a randomized clinical trial has shown a reduction in mortality. Lessons learnt during the COVID-19 pandemic will be of utmost importance for future pandemics.

## 1. Introduction

In December 2019, the coronavirus disease (COVID-19) pandemic caused by the Severe Acute Respiratory Syndrome Coronavirus 2 (SARS-CoV-2) provoked an unprecedent health and social crisis worldwide. Three years later, on 5 May 2023, after more than 7,000,000 confirmed deaths, the World Health Organization (WHO) declared that COVID-19 was no longer a Public Health Emergency of International Concern (PHEIC). The time elapsed is enough to make some final considerations regarding the various therapies employed to fight COVID-19, such as the plasma collected from recovered individuals named COVID-19 convalescent plasma (CCP), which has, for more than one year (January 2020–March 2021), represented the only specific, antibody-based passive immunotherapy available against this potentially life-threatening viral infection. Among anti-COVID-19 therapies, CCP has been the most extensively studied, its safety and efficacy being assessed by more than 30 randomized controlled trials (RCTs) so far, whose results have been fully published [1,2].

The aim of this narrative review is to critically summarize the role of CCP against COVID-19 and evaluate all of its aspects, from collection to clinical use.

## 2. Search Methods

A literature search through MEDLINE and PubMed electronic databases was performed for publications during the period from 1 December 2019 to 31 August 2023 using the following Medical Subject Heading (MeSH) and keywords: “COVID-19”, “SARS-CoV-2”, “COVID-19 convalescent plasma”, “hyperimmune plasma”, “passive immunotherapy”, “therapy”, “hospital”, “outpatients”, “ambulatory”, “safety”, “randomized controlled trials” and “ABO blood type”. We also screened the reference lists of the most relevant review articles for additional studies not captured in our initial literature search.

## 3. Convalescent Plasma against COVID-19

### 3.1. Collection, Validation and Characteristics of CCP

CCP has generally been collected by single productive plasmapheresis, which is quite expensive and requires trained personnel, although whole blood donation and HemoClear^®^ procurement have also been reported in low-to-middle income countries [3]. In the initial phase of the pandemic, qualification was bound to viral neutralization tests to measure neutralizing antibody (nAb) titers, which are time- and money-consuming, lack standardization, and can only be run in biosafety level 3 facilities [4]. It was later proved that high-throughput automated serology had excellent correlations with nAbs titers and could provide a cheaper and faster solution. Several countries also initially mandated accessory NAT testing (e.g., parvovirus B_19_, HAV, and HEV) and pathogen reduction technologies (i.e., photoinactivation including methylene blue + visible light, riboflavin + ultraviolet B or amotosalen + ultraviolet A). Both were unavailable at transfusion facilities at the beginning of the pandemic, are time- and money-consuming, and the latter was also discovered to be potentially antibody-disrupting [5,6]. Despite the fact that no transfusion-transmitted coronavirus infection had been ever documented at that time (as expected for a virus with a low-grade and transient viremic stage), the fact that the donors with the highest nAb titers were those discharged after hospitalizations [7] suggested such an excess of prudence. Both measures were no longer mandated since 2021, leading to highly simplified collections. At that time, the mass vaccination campaign combined with the unrestricted wave of infections had led to an unprecedently high prevalence of high nAb titer donors regardless of symptom severity. It has been shown that such hybrid immunity preserves efficacy against current and future variants, provided collections are restricted to the deciles with the highest nAb titers [8,9]. In addition, the widespread diffusion of SARS-CoV-2 infection among the population has permitted the collection of VAX-plasma (i.e., CCP collected from individuals vaccinated and recently recovered from COVID-19) exclusively from regular blood donors, thus further enhancing the CCP safety against known and unknown blood-borne pathogens. This excess availability has paradoxically not been exploited in most countries, at a time when the number in need had dramatically reduced. While in the US CCP has been regulated and strictly defined by the FDA, CCP still remains a blood component without a specific definition in the EU EDQM manuals, with storage conditions overlapping fresh frozen plasma used for coagulopathy [10]: this urgently requires an update since antibodies, as opposed to labile clotting factors, have been shown to remain stable at room temperature for months [11], further simplifying storage of CCP. CCP qualification also still has margins of improvement, and qualification for anti-spike IgA and IgM, anti-nucleocapsid antibodies, or other mediators could add value [12].

### 3.2. Safety of CCP

As previously stated, much of the additional qualification tests initially mandated for CCP were proposed on the basis of additional infectious safety concerns, which have proven unfounded so far.

Additional concerns were initially raised, such as antibody-dependent enhancement (ADE) of viral infection and transfusion reactions. All of those concerns have remained theoretical so far, and several systematic reviews and metanalysis have found no increased risk compared to transfusion of fresh frozen plasma [13,14]. An additional concern is the occurrence of venous and arterial thrombotic adverse events related to CCP transfusion, an issue not so trivial considering the highly prothrombotic context of COVID-19 and the presence of procoagulant factors in fresh frozen plasma [12]. A recent systematic review and meta-analysis of 39 RCTs enrolling nearly 24,000 participants did not find an increased incidence of thromboembolic complications in CCP-treated patients versus controls [15], reassuring definitively the safety profile of CCP against the thrombotic risk.

### 3.3. Efficacy of CCP in Outpatients

A recent metanalysis comparing the efficacy of CCP with other outpatient regimens has shown that CCP is only slightly inferior to authorized anti-spike monoclonal antibodies (mAbs) and small-molecule antivirals [16]. Considering that anti-spike mAbs were all deauthorized in 2022–2023 because of the loss of baseline activity against recent Omicron sublineages (mostly due to convergent evolution at critical amino acid residues [17]), CCP remains the only passive immunotherapy available for frail patients who have contraindications or cannot tolerate the toxicities of small-molecule antivirals. Furthermore, even until baseline anti-spike mAb efficacy was preserved, CCP has been shown to rescue treatment-emergent mAb escape [18]. It is nevertheless out of discussion that nowadays, the advent of oral small molecule antivirals represents a robust alternative to CCP [19]. It must be stressed, however, that in many European countries, CCP use has never been authorized (not even at an emergency level like in the US), therefore it has always remained an experimental product only for in-hospital use within ethical committee-approved protocols. Such a decision has irremediably damaged CCP efficacy, favoring studies on late hospital use instead of those on early, and more appropriate, ambulatory use. Thus, proper RCTs on CCP use in outpatients were conducted only in a late pandemic phase [20], following the demonstration of the efficacy of the early use of monoclonal antibodies against SARS-CoV-2.

### 3.4. Efficacy of CCP in Hospitalized Patients

In single-agent, placebo-controlled RCTs, delayed CCP use has generally been associated with no benefit [1,2]. However, a re-evaluation of literature data discovered signals of CCP efficacy in many unfavorable RCTs, after the analysis of subgroups of patients receiving early and high-titer CCP treatment [1]. In addition, a recent systematic review has nevertheless shown a 13% reduced mortality when CCP is administered within 5 days since hospitalization [21]. This could be in part related to the anti-inflammatory effects of non-antibody ingredients within CCP [12], as well as to some of the hospitalized patients being partly immunocompromised and with persistent seronegativity. The multicenter Italian TSUNAMI (the Transfusion of Convalescent Plasma for the Early Treatment of Patients with COVID-19) trial, which was conducted in 487 hospitalized COVID-19 patients, was published in November 2021, but its negative results on CCP efficacy were anticipated in international press in April 2021. This RCT had a detrimental effect on CCP [22] for at least two reasons: first, it caused the stop of CCP use (and therefore the collection), not only in Italy but also in most Western countries. Secondly, it ignored important signals of CCP efficacy in a subgroup of patients with moderate COVID-19, which should have pushed the design of a further trial conducted, more appropriately, in patients with milder disease.

With molnupiravir deauthorized by the EMA because of the lack of efficacy in vaccinated cases and potential mutagenicity [23], only Paxlovid^®^ and remdesivir are left as alternatives: the prevalence of contraindications to the latter is particularly high in hospitalized patients [24]. Thus, the paucity of antiviral treatments available against COVID-19 and the lack of CCP collection and availability has created a therapeutic hole, which is particularly dangerous for fragile immunocompromised patients, who, not uncommonly, are unresponsive to vaccines and have comorbidities that hamper the use of antivirals.

### 3.5. Efficacy of CCP in Immunocompromised Patients

Persistent SARS-CoV-2 infection remains a major healthcare concern for immunocompromised patients. This is especially relevant for onco-hematological patients. Systematic reviews have shown CCP efficacy in both primary [25] and secondary immunodeficiencies [26]. The duration of treatment is currently under discussion, with repeated infusion likely required for maintenance until eradication [27]. Importantly, no antiviral has been specifically tested in RCTs in immunocompromised patients, except for a single RCT employing high-titer VaxCCP [28], which has shown a significant reduction in mortality in onco-hematological patients. A growing number of scientific societies and regulatory authorities around the world are acknowledging such efficacy by recommending CCP in their guidelines [29,30], except for WHO not having issued an update since December 2021 [31]. Similarly, Cochrane reviews still persist at issuing negative recommendations ignoring subgroup analyses [32].

### 3.6. Greys Zones

An exact therapeutic dose of anti-spike antibodies in CCP therapy remains undefined: it is believed to result from the combination of nAb titers, cumulative CCP volume, and body weight of the recipient, but antibody affinity can also play a role [33].

The role of many more spike proteins is increasingly being acknowledged, which could require qualifying the CCP units for different additional biomarkers. Another interesting field of investigation is the role of the ABO antigens in infection, disease progression, and long-COVID incidence, as well the role of anti-A isoagglutinin as a mediator of therapeutic efficacy [34,35]. Possible molecular mechanisms of such effects include both the ability of spike to bind to A antigens, and the ability of anti-A antibodies at neutralizing the virions. Overall, the analysis of the published literature data shows that individuals with blood group O and those with group A are at lower and increased risk of becoming infected by SARS-CoV-2, respectively. In contrast, no conclusive evidence is available regarding the association between ABO blood group and COVID-19 severity and outcomes [34]. No study has been reported yet on the association between ABO blood groups and post-acute sequelae of COVID-19. Further studies are also needed to verify whether in COVID-19 clinical responses to plasma-derived antibody-based treatments (i.e., convalescent plasma and polyclonal IgGs) are driven, in addition to the levels of neutralizing antibodies, also by the presence of natural occurring ABO antibodies. In our opinion, however, the most intriguing area of development will be combined therapies with other small-molecule antiviral drugs. This has been poorly investigated so far, while being of utmost importance for immunocompromised patients with persistent infections [36].

Finally, delivery of CCP into the respiratory mucosae of the upper respiratory tract, which represents the entry door for SARS-CoV-2, is worth investigating in early outpatients, granting higher safety and lower therapeutic doses [37].

### 3.7. Considerations for Next Pandemic

At the end of the pandemic emergency, the world is still lacking guidelines from regulatory authorities [38] for deploying and designing RCTs employing convalescent plasma in future pandemics. As previously discussed, the poor design of RCT during the first year of the COVID-19 pandemic has hindered the final success of this effective treatment, and such emotional mistakes (e.g., delivering CCP units with a nAb titer lower than the one occurring in recipients before transfusions [39]) should be carefully avoided in the future [1,2]. It will be of utmost importance that regulatory authorities and institutions economically support investigators, even for second-generation RCTs. Data sharing should be maximized, which has not always been the case during this pandemic [40].

## 4. Conclusions

Three years into the COVID-19 pandemic, we can conclude that CCP is a fully safe and effective treatment for both outpatients and inpatients, does not impact the self-sufficiency of the plasma derivatives, and is extremely effective in seronegative patients with persistent infections. Further RCTs are needed to assess CCP in combination regimens. It remains to be established whether, at the steady state of viral evolution, concentrated polyclonal IgG formulations would be as equally effective as CCP [41,42].

## Data Availability

This manuscript did not generate any dataset.
